# Complete Genome Sequence of *Weissella confusa* LM1 and Comparative Genomic Analysis

**DOI:** 10.3389/fmicb.2021.749218

**Published:** 2021-09-28

**Authors:** Shenglei Yuan, Yundan Wang, Fangqing Zhao, Le Kang

**Affiliations:** ^1^State Key Laboratory of Integrated Management of Pest Insects and Rodents, Institute of Zoology, Chinese Academy of Sciences, Beijing, China; ^2^CAS Center for Excellence in Biotic Interactions, University of Chinese Academy of Sciences, Beijing, China; ^3^Beijing Institutes of Life Science, Chinese Academy of Sciences, Beijing, China; ^4^College of Life Science, Hebei University, Baoding, China

**Keywords:** *Locusta migratoria*, gut bacteria, *Weissella confusa*, comparative genomics, pan-genome

## Abstract

The genus *Weissella* is attracting an increasing amount of attention because of its multiple functions and probiotic potential. In particular, the species *Weissella confusa* is known to have great potential in industrial applications and exhibits numerous biological functions. However, the knowledge on this bacterium in insects is not investigated. Here, we isolated and identified *W. confusa* as the dominant lactic acid bacteria in the gut of the migratory locust. We named this strain *W. confusa* LM1, which is the first genome of an insect-derived *W. confusa* strain with one complete chromosome and one complete plasmid. Among all *W. confusa* strains, *W. confusa* LM1 had the largest genome. Its genome was the closest to that of *W. confusa* 1001271B_151109_G12, a strain from human feces. Our results provided accurate evolutionary relationships of known *Weissella* species and *W. confusa* strains. Based on genomic analysis, the pan-genome of *W. confusa* is in an open state. Most strains of *W. confusa* had the unique genes, indicating that these strains can adapt to different ecological niches and organisms. However, the variation of strain-specific genes did represent significant correlations with their hosts and ecological niches. These strains were predicted to have low potential to produce secondary metabolites. Furthermore, no antibiotic resistance genes were identified. At the same time, virulence factors associated with toxin production and secretion system were not found, indicating that *W. confusa* strains were not sufficient to perform virulence. Our study facilitated the discovery of the functions of *W. confusa* LM1 in locust biology and their potential application to locust management.

## Introduction

The bacterial genus *Weissella* was first established in 1993 and isolated from fermented sausages. It is a member of the well-known group of lactic acid bacteria (LAB) (Collins et al., 1993). *Weissella* are Gram-positive, catalase-negative, and non-endospore-forming cells with coccoid or rod-shaped morphology, belonging to the family Leuconostocaceae of phylum Firmicutes.

Species and strains of *Weissella* have been isolated from various ecological niches and have different functions. They have been isolated from various fermented foods, such as yoghurt, sourdough, and kimchi (Wang et al., 2011; Zannini et al., 2018; Mun and Chang, 2020; Valerio et al., 2020), and play important roles in the process of fermentation, thereby influencing the texture and taste of foods. They also exist in the guts of humans and vertebrate animals, such as giant pandas and rainbow trout (Xiong et al., 2019; Mortezaei et al., 2020; Wang et al., 2020), their functions involve reducing depressive-like behavior (Sandes et al., 2020), influencing gut permeability and intestinal epithelial regeneration (Prado et al., 2020), killing harmful bacteria (Dey and Kang, 2020; Lee et al., 2020; Pelyuntha et al., 2020), affecting host metabolism (Elshaghabee et al., 2020), and preventing cancer cell proliferation (Le et al., 2020). Some *Weissella* species may be beneficial to plants because they inhibit the infection of plant pathogenic fungi, such as *Fusarium verticillioides* (Quattrini et al., 2020).

Recently, more studies suggested that *Weissella* species and strains can be used in the food and pharmaceutical industries. Some *Weissella* species have elicited research interest because of their probiotic, biotechnological, and bacteriocinogenic potential. In particular, some studies have clarified that *W. confusa* can be used as a direct-fed microbial candidate (Sturino, 2018; Cupi and Elvig-Jørgensen, 2019; Bourdichon et al., 2021) and exist in the breast milk of healthy women (Martin et al., 2007; Albesharat et al., 2011). *W. confusa* has been added to the International Dairy Federation (IDF) Inventory (Bourdichon et al., 2012), and the Senate Commission on Food Safety (SKLM) also validated its usage in traditional methods of food fermentation (Vogel et al., 2011).

With the rapid development of sequencing techniques, the genomes of diverse bacterial strains have been sequenced. This advancement promoted research on comparative genomics, which helped illustrate the relationships between genotypes and phenotypes (Wang et al., 2018), thereby addressing the effects of isolation source on the genetic variations (Mao et al., 2021), revealing horizontal gene transfer (Singh et al., 2021), and performing taxonomic revision (Lavecchia et al., 2021). As the importance of *Weissella* species has been recognized, attempts have been made to explore their evolutionary relationship and genomic characteristics. Comparative genomic analyses of some *Weissella* species, such as *Weissella hellenica* (Panthee et al., 2019) and *Weissella cibaria* (Li et al., 2017), have been conducted. By 2021, 38 genome assemblies of *W. confusa* strains have been deposited into the National Center for Biotechnology Information (NCBI) public database, including 3 complete genomes and 35 draft genomes, and most of them were sequenced in the past 3 years. Comparative genomic analysis of these *W. confusa* strains will allow us to gain a deeper understanding of *W. confusa* species and facilitate the potential application of *W. confusa* strains.

In this study, we identified a strain of *W. confusa* from the gut of the migratory locust (*Locusta migratoria*) and completed its whole-genome sequence. We designated the special strain as *W. confusa* LM1, which stably existed in the gut of the migratory locust, a worldwide locust species (Guo et al., 2020). By performing comparative genomic analysis of *Weissella* species, we obtained a more accurate evolutionary relationship and the genetic characteristics of different *W. confusa* strains. Our results provided important insights into the role of this strain in locust biology and offered a potential target for locust control.

## Materials and Methods

### Bacterial Strain

The bacterial strain used in this study was isolated from the gut of the migratory locust, which was reared as an animal model system at the Institute of Zoology, Chinese Academy of Sciences, Beijing, China. The whole guts of 10 locusts were carefully dissected with sterile devices, homogenized in 10-ml of sterile phosphate-buffered saline (PBS) solution (pH 7.4), and then diluted by sterile PBS solution (1:10,000). Subsequently, 50 ml of the diluted homogenized solution was spread over de Man Rogosa and Sharpe (MRS) medium (Man et al., 1960). A single colony was used to prepare the broth and establish the glycerol stocks. The single colony was also used for 16S rRNA gene amplification by using the universal primers 27F (5′-AGAGTTTGATCCTGGCTCAG-3′) and 1492R (5′-TACGGCTACCTTGTTACGACTT-3′). Amplified products were sequenced and assigned to taxa based on NCBI public databases by using the basic local alignment search tool. Isolated strains were maintained at −80°C as frozen stock cultures in MRS broth.

### DNA Extraction and Whole-Genome Sequencing

*Weissella confusa* LM1 was cultured in MRS broth at 37°C for 12 h. Genomic DNA was extracted by using Qiagen DNeasy Blood & Tissue Kit (Qiagen) according to the instructions of the manufacturer. Harvested DNA quality was checked by agarose gel electrophoresis (0.8%), and DNA quantity was determined by using NanoDrop 1000 (Thermo Fisher Scientific). The whole-genome sequence of *W. confusa* LM1 was obtained through single-molecule real-time (SMRT) sequencing by using PacBio Sequel platform and through next-generation sequencing (NGS) by using Illumina NovaSeq platform. Libraries for SMRT sequencing were constructed with an insert size of 10 kb by using the SMRTbell Template kit (Pacific Biosciences). Libraries for NGS were constructed with an insert size of 350 bp by using Ultra DNA Library Prep Kit (NEB).

### Genome Assembly

Preliminary assembly sequences were obtained on the basis of the raw data from PacBio Sequel platform by using the assembly software PacBio SMRT Link V5.0.^1^ Low-quality reads of less than 500 bp were filtered to obtain clean data and to ensure the accuracy of the subsequent analyses. The automatic error correction function of SMRT portal was performed. Long reads of over 6,000 bp were selected as the seed sequence, the other reads were aligned to the seed sequence by using Basic Local Alignment with Successive Refinement (BLASR) to improve the accuracy of the seed sequence (Chaisson and Tesler, 2012). After obtaining the initial assembly result, the arrow algorithm was used to correct the variant sites in the initial assembly result by using the variant Caller module of the SMRT Link. NGS data were used to further correct the preliminary assembly sequence by BWA (Li and Durbin, 2009). Cyclization was then confirmed according to overlap (at least 1.5 kb). The initiation site was corrected by BLAST with the DnaA genes and confirmed through GC-Skew.

### Gene Prediction and Genome Annotation

The genome of *W. confusa* LM1 was automatically annotated using the GeneMarkS-2+ based on the NCBI Prokaryotic Genome Annotation Pipeline (PGAP) (Tatusova et al., 2016). Each gene was assigned to a category within the Clusters of Orthologous Groups (COG) database (Galperin et al., 2021). Circular layouts were generated using Circos V0.69^2^ (Krzywinski et al., 2009). Ribosome RNA (rRNA) was identified by using RNAmmer V1.2 (Lagesen et al., 2007), and tRNA genes were predicted by tRNAscan-SE V2.0 (Lowe and Chan, 2016). Carbohydrate-Active enZYmes (CAZy) database (Lombard et al., 2014) was used for carbohydrate metabolism analysis by using DIAMOND with a cutoff e-value of 1e-5 and minimum identity of >40% (Buchfink et al., 2015). The online server of the Antibiotics and Secondary Metabolite Analysis Shell (antiSMASH V6.0^3^) was used for the annotation and analysis of secondary metabolite biosynthesis gene clusters with “relaxed” detection strictness (Blin et al., 2021).

### Pathogenicity and Antibiotic Resistance Analysis

The virulence factor database (VFDB) core dataset (setA) was used to curate information about the virulence factors of bacterial pathogens by using DIAMOND with a cutoff e-value of 1e-5 and 70% identity based on the protein data (Chen et al., 2012). Based on the genome data, PathogenFinder V1.1^4^ was used to predict the pathogenicity of the bacteria toward human hosts (Cosentino et al., 2013). Prophage regions of *W. confusa* strains were detected by using the PHASTER server (Arndt et al., 2016). Phylogenetic tree of intact phages was created by using VICTOR^5^ (Meier-Kolthoff and Goker, 2017). ResFinder database was used to identify the acquired antibiotic resistance genes based on genome data by using the ResFinder 4.1^6^ with thresholds of 90% identity and 60% gene coverage (Zankari et al., 2012). The Comprehensive Antibiotic Resistance Database (CARD) was also used to identify antibiotic resistance genes by using the Resistance Gene Identifier (RGI) software to predict resistome from protein data based on homology and SNP models with the selection criteria of perfect and strict hits only^7^ (Alcock et al., 2020).

### Comparative Analysis

For the comparative genome analysis, the genomic sequences of each *Weissella* species and strains were downloaded from NCBI. Average nucleotide identity (ANI) value was calculated by using JSpecies V1.2.1 with BLAST (Richter and Rossello-Mora, 2009) under the default parameters (Goris et al., 2007). The dropoff value for gapped alignment was set to 150, the e-value was set to 1e-15, the identity was set to 30%, and alignment length was set to 1,020 bp. ANI is the average value based on the comparisons of all orthologous protein-encoding genes of pairwise genomes and is a classic method for judging whether strains belong to the same species. Strains with ANI values of over 95% are generally considered to be of the same species (Yoon et al., 2017). The obtained ANI value matrix was clustered and visualized by using R package pheatmap V1.0.12. Multiple genomic sequence alignment visualization was displayed by using Mauve V20150226 (Darling et al., 2004), which is used for multiple genome alignments to identify genome-wide rearrangements and inversions. Minimum locally colinear block (LCB) weight was set as 15 and Muscle V3.6 was used for full alignment (Edgar, 2004).

### Pan-Genome Analysis

The pan-genome of *W. confusa* was constructed by using Bacterial Pan Genome Analysis (BPGA) V1.3.0 with 50% sequence identity cu-off for clustering (Chaudhari et al., 2016). Phylogenetic tree was constructed on the basis of the core genes of different strains through the neighbor-joining method. The effects of isolation ecological niches on the genetic characteristics of *W. confusa* strains were analyzed via principal component analysis (PCA) according to the gene presence–absence matrix generated from BPGA.

### Data Availability

The complete genomic sequences of *W. confusa* LM1 have been deposited in GenBank under the accession numbers CP080582.1 (chromosome) and CP080583.1 (plasmid). The BioProject accession number for this project was PRJNA752246 (http://www.ncbi.nlm.nih.gov/bioproject/752246).

## Results

### *Weissella confusa* LM1 Strain and Its Genome

We used the MRS medium to screen and isolate LAB in the gut of the migratory locust. Approximately 10^7^ colony-forming units (CFUs) were found in the whole gut of locusts. Eighty colonies were randomly selected to identify species composition by sequencing 16S rRNA gene sequence. We found that 74 (92.5%) of the colonies were *W. confusa*, which stably existed in the locust gut.

**FIGURE 1:**
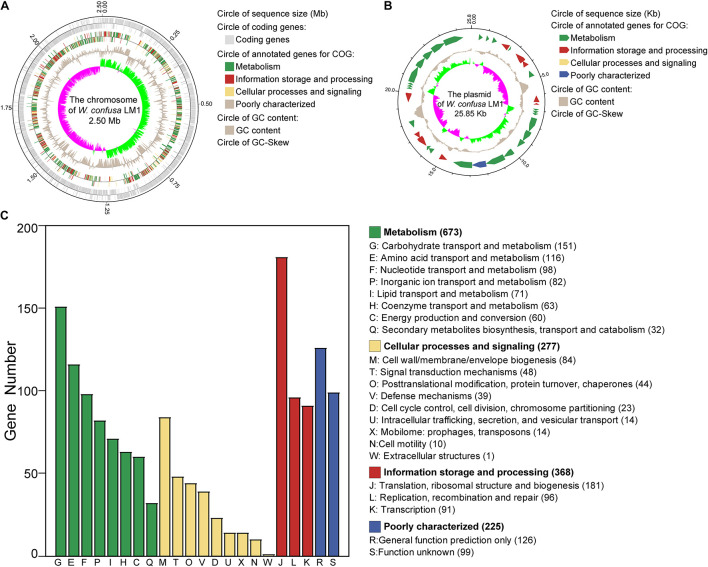
Genome analysis of *Weissella confusa* LM1. **(A)** The chromosome features of *W. confusa* LM1. **(B)** The plasmid features of *W. confusa* LM1. **(C)** Clusters of Orthologous Groups (COG) analysis of *W. confusa* LM1 genome.

We isolated the strain and sequenced the whole genome of *W. confusa* LM1. The *W. confusa* LM1 genome consisted of a circular chromosome and a circular plasmid (Figures 1A,B). The size of its genome was 2.53 Mb; the sizes of the circular chromosome and circular plasmid were 2.50 Mb and 25.85 kb, respectively (Table 1). A total of 2,320 protein-coding sequences (CDSs) were predicted by GeneMarkS, and 1,543 genes were annotated in the COG database, which were primarily mapped to the metabolism category, particularly carbohydrate transport and metabolism (Figure 1C). This strain from locust had 114 RNA genes, which are as follows: 84 tRNAs, 10 5S rRNA, 9 16S rRNA, 9 23S rRNA, and 2 sRNA genes.

**TABLE 1:** General features of *Weissella confusa* LM1 genome.

Type	Characteristic
Genome size	2,529,433 bp
Chromosome number	1
Plasmid number	1
Chromosome length	2,503,583 bp
Plasmid length	25,850 bp
GC content (%)	44.4
CDS number	2320
tRNA number	84
5s rRNA number	10
16s rRNA number	9
23s rRNA number	9
sRNA number	2

### Comparative Genomics of *Weissella*

**FIGURE 2:**
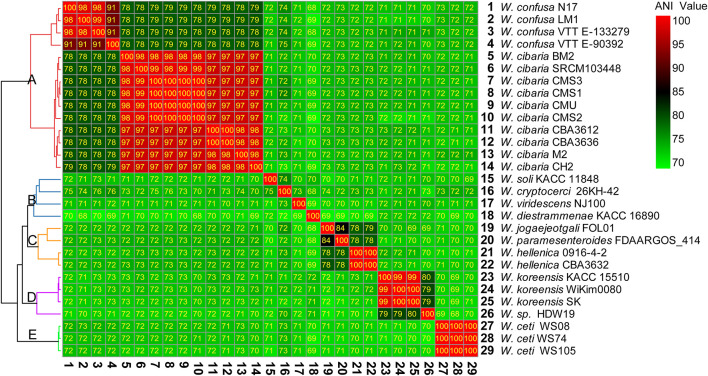
Heatmap of percentage average nucleotide identity (ANI) value between the different species and strains of *Weissella*. The strain on the vertical axis serves as the reference.

There were 28 complete genomes of *Weissella* genus in the NCBI public databases. Based on the complete genome sequences, we calculated the ANI value between different species and strains (Figure 2). The ANI value reflected the evolutionary relationship between two species, and 95% sequence identity was considered as the species level threshold.

The 29 complete genome assemblies of *Weissella* were clustered into five clusters, namely, A, B, C, D, and E (Figure 2). Cluster A had 2 species and 14 strains of *W. cibaria* and *W. confusa*, whose ANI values were about 78–80% compared with each other. However, the ANI value was less than 75% compared with that of other species. Cluster B had four species and four strains, namely, *Weissella soli*, *Weissella cryptocerci*, *Weissella viridescens*, and *Weissella diestrammenae*, and all of them had lower ANI values compared with other strains. In particular, the ANI value of *W. diestrammenae* was only about 70% compared with that of other species, indicating its species-specific status. Cluster C had three species and four strains, namely, *Weissella jogaejeotgali*, *Weissella paramesenteroides*, and *W. hellenica*, and they were found to have a close evolutionary relationship with an ANI value of above 78%. In particular, the ANI value between *W. jogaejeotgali* and *W. paramesenteroides* was 84%. Cluster D had two species and four strains, namely, *Weissella koreensis* and the unidentified species *W.* sp. HDW19. The species of *Weissella* sp. HDW19 had a high ANI value of 80% with *W. koreensis*. Cluster E had 1 species, *W. ceti*, and three strains. *W. ceti* had a low ANI value with all other species.

### Comparative Genomics of *Weissella confusa*

By 2021, three complete and 35 draft genomes of *W. confusa* strains were deposited into the NCBI public database, and 85% of them were sequenced in the past 3 years. According to the information on source and region, *W. confusa* could be isolated from different niches, such as food, plants, and animals, especially in vertebrate animals (Supplementary Table 1). LM1 was the first insect-derived *W. confusa* strain to be sequenced. It had the largest genome size (2.53 Mb), which was at least 6% more than those of the other strains (2.18–2.38 Mb). Additionally, LM1 had a lower GC content than the other *W. confusa* strains.

**FIGURE 3:**
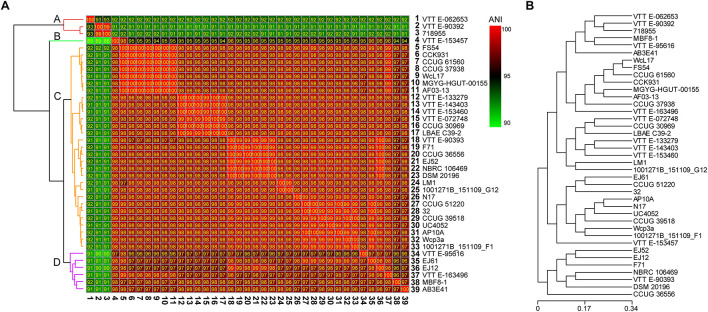
Evolutionary relationships of 39 *W. confusa* strains. **(A)** Heatmap of percentage ANI value among different *W. confusa* strains; the strain on the vertical axis serves as the reference. **(B)** Evolutionary tree analysis based on the protein-coding genes.

To analyze the evolutionary relationships among different *W. confusa* strains, we calculated the ANI value according to the genomic information of 39 strains (Figure 3A) and performed an evolutionary tree analysis based on the CDSs (Figure 3B). Although all strains were named *W. confusa*, according to ANI values, the ANI values fluctuated between 90 and 100%. According to their ANI values. The 39 strains were classified into four clusters, namely, A, B, C, and D, on the basis of their genome sequences (Figure 3A). Cluster A had 3 strains, which had a relatively low ANI value compared with the 36 other strains (91–92%). The *W. confusa* VTTE-062653 was remarkably different from all the other strains. However, the ANI value between *W. confusa* VTTE-90392 and *W. confusa* 718955 was 99%. Cluster B had 1 strain, *W. confusa* VTTE-153457, whose ANI value was approximately 95% compared with those of other strains. Cluster C had 29 strains, and they all had high ANI values (98–100%). Cluster D had six strains, whose ANI values were in the range of 96–98%. *W. confusa* LM1 was the most similar to *W. confusa* 1001271B_151109_G12, which was isolated from human feces (United States).

**FIGURE 4:**
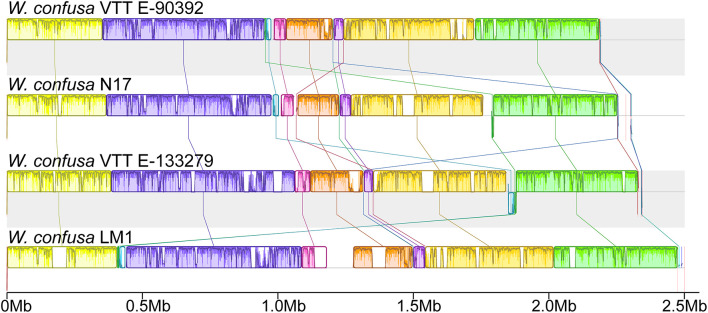
Complete genome alignment of *W. confusa* strains. MAUVE alignment of the genome sequences of *W. confusa* VTTE-90392, *W. confusa* N17, *W. confusa* VTTE-133279, and *W. confusa* LM1. Boxes in the same color represent homologous regions [local colinear blocks (LCBs)] between *W. confusa* genomes. Uncolored regions within the LCBs or in-between LCBs indicate the presence of specific sequences in this strain.

To analyze the genomic structures among different *W. confusa* strains, we compared all four complete genomes and subjected them to similarity analysis by using the Mauve software (Figure 4). With *W. confusa* LM1 as the reference, the four genomes exhibited 11 LCBs, and *W. confusa* LM1 showed more uncolored regions.

### Pan-Genome Analysis of *Weissella confusa*

**FIGURE 5:**
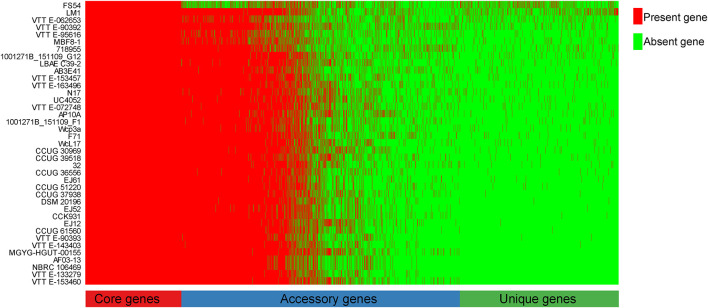
Gene distribution of 39 *W. confusa* strains based on the gene presence–absence matrix generated from the Bacterial Pan Genome Analysis (BPGA).

**FIGURE 6:**
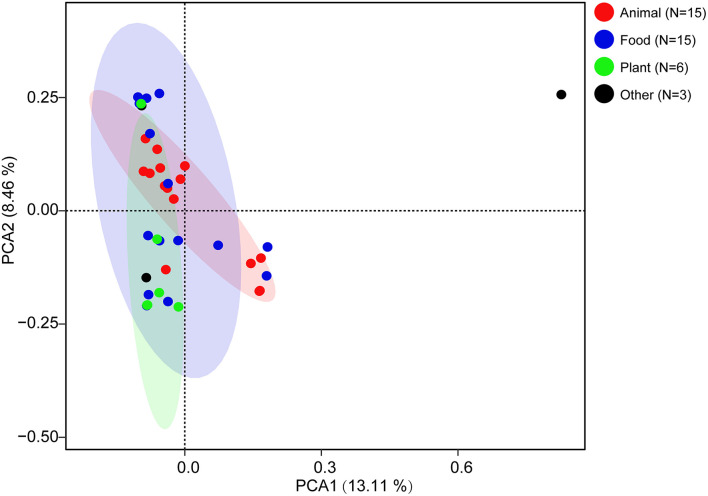
Effects of ecological niches on the genomic characteristics of *W. confusa* strains.

**FIGURE 7:**
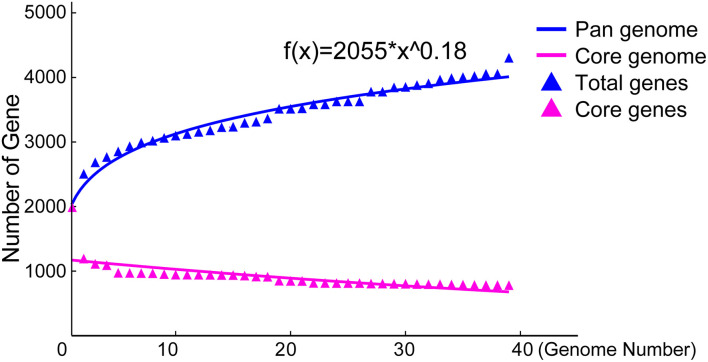
Pan- and core-genome plots of *W. confusa* based on the results of pan-genome analysis of 39 *W. confusa* strains.

To explore the whole genetic characteristics of *W. confusa*, we performed pan-genome analysis and obtained the core, accessory, and unique genes of the 39 strains of *W. confusa* (Figure 5). The 768 genes shared by all *W. confusa* strains formed the core genome. The 2,246 genes present in more than one strain formed the accessory genome, and 1,193 genes specific to one single strain formed the unique genome. The percentage of unique genes was divergent in different *W. confusa* strains (Figure 5). Some strains, such as *W. confusa* FS54 (32%), *W. confusa* LM1(11%), and *W. confusa* VTTE-062653 (7%), contained a high percentage of unique genes. By contrast, five strains, including *W. confusa* MGYG-HGUT-00155, *W. confusa* AF03-13, *W. confusa* NBRC 106469, *W. confusa* VTTE-133279, and *W. confusa* VTTE-153460, did not have unique genes. These different *W. confusa* strains were widely distributed in food, animals, and plants. However, little was known about the relationship between their genomic characteristics and their ecological niches. Among the 39 *W. confusa* strains, 15 strains were isolated from food, 15 strains were isolated from animals, 6 strains were isolated from plants, and 3 strains were isolated from other ecological niches (Supplementary Table 1). On the basis of the gene presence–absence matrix of different *W. confusa* strains, no close relationship was found between their genetic characteristics and ecological niches (Figure 6). Moreover, we performed the fitting curve f(x) = a × x^b (a = 2,055, b = 0.18) based on the correlation of total gene number and genome number. The curve parameter indicated that the genome of *W. confusa* was still open (Figure 7). The open pan-genome implied a great potential for discovering novel genes with the increasing number of *W. confusa* strains sequenced.

**FIGURE 8:**
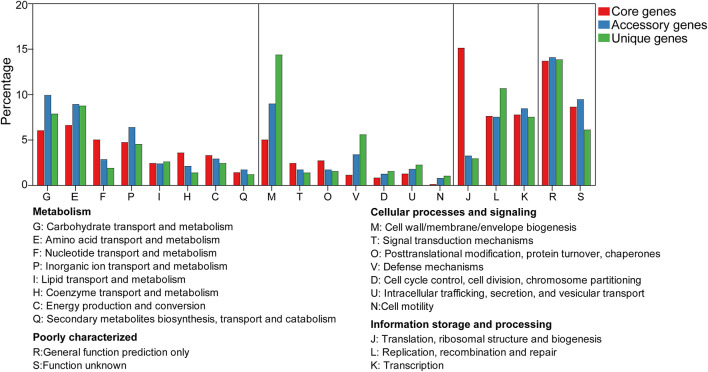
Distribution of core, accessory, and unique genes on the COG category.

To compare genome functional categories, we used functional assignments from the COG database. In the pan-genome of *W. confusa*, we found that most core, accessory, and unique genes fell in the metabolism category (Figure 8). Meanwhile, genes with predicted functions and unknown functions were also more abundant (Supplementary Figure 1). Compared with the core genome, the majority of genes in the no-core genome (accessory and unique genomes) were involved in carbohydrate transport and metabolism (G), amino acid transport and metabolism (E), cell wall/membrane/envelope biogenesis (M), defense mechanisms (V), and cell motility (N). By comparison, the vast majority of genes in the core genome were involved in housekeeping functions, such as nucleotide transport and metabolism (F), coenzyme transport and metabolism (H), and translation, ribosomal structure, and biogenesis (J).

**FIGURE 9:**
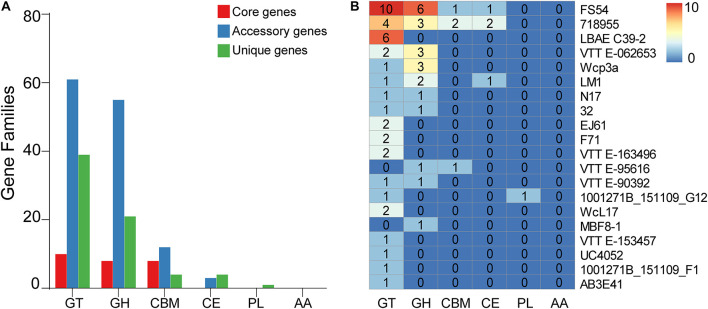
Distribution of pan-genome of *W. confusa* on CAZymes. **(A)** The counts of orthologous genes assigned in the core genome, accessory genome, and unique genome on CAZymes. **(B)** The counts of unique genes of different *W. confusa* strains on CAZymes.

To further understand the carbohydrate metabolism in *W. confusa*, we identified the CAZymes based on the pan-genome. Most genes fell into glycosyltransferases (GTs), glycoside hydrolases (GHs), carbohydrate esterases (CEs), carbohydrate-binding molecules (CBMs), whereas a few genes were annotated in polysaccharide lyases (PLs). All of the genes were not annotated in auxiliary activities (AAs). A few core genome genes were on CAZymes. Compared with the core genome, the accessory genome had more genes on GT, GH, CBM, and CE, the unique genome had more genes on GT, GH, and CE, but fewer genes on CBM (Figure 9A). In addition, different *W. confusa* strains exhibited various numbers of CAZyme genes (Supplementary Table 2). There were 110 CAZyme genes in *W. confusa* 718955, but only 85 CAZyme genes in *W. confusa* MBF8-1. This finding implied that the metabolic abilities of carbohydrates were specialized among *W. confusa* strains. To further compare the specificity of different strains, we analyzed the CAZyme genes of different strains based on unique genome (Figure 9B). Twenty *W. confusa* strains contained unique CAZyme genes, and GT (56%) and GH (31%) genes were the most common in the unique CAZyme genes, such as GT2 (28%), GT4 (17%), GH23 (7%), and GH70 (6%).

### Secondary Metabolite in *Weissella confusa*

The genomic repertoire for the secondary metabolite synthesis of the 39 *W. confusa* strains was assessed by using antiSMASH. *W. confusa* strains were found to contain a small number of genes that encode key secondary metabolism biosynthesis enzymes. Only 42 gene clusters encoded secondary metabolites in all the 39 *W. confusa* strains (Supplementary Table 3). Moreover, 42 gene clusters that encoded secondary metabolites were classified into three types, namely, T3PKS (39), arylpolyene (2), and lassopeptide (1). Each strain harbored one T3PKS. The strain *W. confusa* FS54 and *W. confusa* LM1 harbored one arylpolyene. The strain *W. confusa* WcL17 harbored one lassopeptide.

### Pathogenicity and Antibiotic Resistance in *Weissella confusa*

Based on the VFDB core dataset (setA), we scanned the *W. confusa* genomes for virulence-related factors. In total, we identified 107 putative orthologs involved in the production of virulence factors in 39 *W. confusa* strains (Supplementary Table 4). These 107 putative orthologs were classified into three virulence-related factors, namely, hyaluronic acid capsule, capsule, and EF-Tu. All *W. confusa* strains had three virulence-related factors except *W. confusa* FS54, which only had one capsule. There was no strain-specific virulence factor. According to the function of the three virulence-related factors (Supplementary Table 4), we found that none of the virulence-related factors was associated with toxin production and secretion system, thereby indicating that *W. confusa* strains were not sufficient to perform virulence. Moreover, we predicted the pathogenicity of *W. confusa* strains toward human hosts by using PathogenFinder. *W. confusa* strains were predicted to be non-human pathogens. We estimated the antibiotic resistance of *W. confusa* strains based on the antibiotic resistance databases, ResFinder and CARD, and no antibiotic resistance genes were recognized in *W. confusa* strains. We detected the phages in 39 genomes of *W. confusa* strains. There were 112 phages, including 40 intact phages, 55 incomplete phages, and 17 questionable phages (Supplementary Table 5). There were 30 *W. confusa* strains with intact phages, which could be divided into two major clusters (Supplementary Figure 2), and we did not find any virulence factors and antibiotic resistance genes in all phages.

## Discussion

LAB are generally recognized as safe microorganisms, and they have many beneficial functions in the host gut (Feord, 2002). In this study, we used the MRS medium to culture LAB from the locust gut. *W. confusa* was found to be a dominant LAB. The *W. confusa* strains have many functions and are distributed in different niches, such as food, plants, and vertebrate animals. However, a few *W. confusa* strains are found in insects, except in locust and cricket (Milanovic et al., 2018; Galli et al., 2020). We isolated the locust-derived *W. confusa* strain, designated it as LM1, and then sequenced its genome. *W. confusa* LM1 had the largest genome size (Supplementary Table 1) and had a high percentage of unique genes (Figure 5), thereby indicating the specificity of locust-derived *W. confusa* and providing more genomic information on the pan-genome of *W. confusa*.

At present, the classification of the bacteria is mostly based on the 16S rRNA gene sequence. However, some species belonging to one genus may have very similar 16S rRNA gene sequences, leading to a poor resolution when distinguishing different species (Case et al., 2007). The ANI analysis with genomic information is helpful in constructing a more precise phylogenetic relationship and performing taxonomic revision. In the present study, *W. confusa* strains VTTE-062653, VTTE-90392, and 718955 were found to have low ANI value (92%) compared with other *W. confusa* strains (Figure 3). This 92% ANI value did not meet the standards of species delineation (95%). Thus, we suggested that these three strains should be treated as new species of *Weissella* or new subspecies of *W. confusa.* Moreover, ANI analysis were helpful in predicting and analyzing the functions of different species and strains. For instance, *W. cibaria* and *W. confusa* were found to have a high ANI value (Figure 2) and possessed many similar characteristics (Fusco et al., 2015; Nel et al., 2019; Rizzello et al., 2019; Quattrini et al., 2020).

The pan-genome status of *W. confusa* is open (Figure 7), as this species has a high percentage of accessory (53%) and unique (28%) genes. Some accessory and unique genes are from lateral gene transfer, which allows different strains of species to adapt better to specific niches (Caputo et al., 2019). The open pan-genome of *W. confusa* was consistent with the fact that *W. confusa* can exist in various niches (Supplementary Table 1). Core genes are important to understand the core factor that maintains the survival and reproduction of this species because they can be used to detect interactions among different species (Yang et al., 2019). The genome of the *W. confusa* LM1 strain and the pan-genome of *W. confus*a were helpful in detecting the interaction between *W. confusa* and the migratory locust.

Comparative genomic analysis revealed a high genomic variation among *W. confusa* strains (Figure 5). This result implied that different strains are able to perform strain-specific function. The strain-specific function would link with the production of exopolysaccharides (EPSs), which are extracellular macromolecules with wide-ranging biofunctional properties and one of the distinctive phenotypic features of *Weissella* (Fusco et al., 2015; Teixeira et al., 2021). Among *Weissella* species, *W. confusa* is one of the most important EPS producers (Teixeira et al., 2021). However, different *W. confusa* strains, like *W. confusa* VP30, XG-3, and KR780676, were found to have various EPSs with different functions (Jin et al., 2019; Kavitake et al., 2020; Zhao et al., 2020). On the other hand, different *W. confusa* strains were found to have property to produce strain-specific bacteriocin. For instance, *W. confusa* A3 can produce bacteriocin A3 (Goh and Philip, 2015), and *W. confusa* MBF8-1 can produce Weissellicin-MBF (Malik et al., 2021). Thus, the strain-specific genes and their function need to further investigate because of EPS and bacteriocin potential applications to the food, medical, and pharmaceutical industries (Valerio et al., 2020).

Some insect pests possessed insecticide resistance for bacteria-mediated degradation (Itoh et al., 2018; Blanton and Peterson, 2020). Exploring the dominant gut bacteria and its potential functions will be helpful in pesticide development, pest management strategies, and pesticide bioremediation. A recent study discovered a new metabolism pathway that the *W. confusa* Lb.Con strain can degrade chlorpyrifos (Hamoud and Sifour, 2021), which is a commercial pesticide used to control foliar insects, including locusts (Zhang et al., 2019). *W. confusa* is a dominant gut bacteria of the migratory locust, high abundance of *W. confusa* may be one of the important reasons for the marginal chlorpyrifos resistance of this pest (Yang et al., 2009). Given that spraying fenitrothion can enhance the abundance of fenitrothion-degrading *Burkholderia* strains in *Riptortus pedestris* gut (Kikuchi et al., 2012), the high level of *W. confusa* in the migratory locust gut is probably the consequence of the increased amount of pesticides or other stresses affecting the migratory locust. These results further indicated that the sustainable management of major insect pests need to consider the role of gut microbiota (Blanton and Peterson, 2020).

As a stable and dominant strain in the migratory locust gut, *W. confusa* LM1 has the potential to be modified into engineered bacteria with specific functions. With the development of genetic technology, engineered symbiotic microorganisms have been used under different conditions. In mammals, many engineered symbiotics can metabolize harmful substances and serve as a vector to treat certain diseases, and some of them are already in the clinical stage of research (Charbonneau et al., 2020). Furthermore, significant progresses have been achieved in the exploration of the effects of engineered strains on some insects. Some examples include the following: modified *Photorhabdus* can enhance the control of the agricultural pest in maize root (Machado et al., 2020); modified *Snodgrassella alvi* can increase the longevity and immunity of beneficial insect (Leonard et al., 2020); and modified *Serratia* can reduce the transmission of malaria by reducing *Plasmodium* in mosquitoes (Wang et al., 2017). The migratory locust is an important agricultural pest, and outbreaks of locust plague impose huge losses to agriculture and animal husbandry (Guo et al., 2020). At present, the control of locust plague primarily depends on chemical agents, which act rapidly but may lead to some ecological problems (Roussi, 2020). The stable and safe symbiotic *W. confusa* LM1 can serve as a potential target for controlling locust plague by inducing this strain to express some metabolites and, thus, decrease the vigorous traits of locust, such as reproduction, flight, and aggregation pheromone. The genomic information of this stable strain in our report lays the foundation for further exploration and application.

## Data Availability Statement

The datasets presented in this study can be found in online repositories. The names of the repository/repositories and accession number(s) can be found below: http://www.ncbi.nlm.nih.gov/bioproject/, PRJNA752246.

## Author Contributions

SY, LK, and FZ conceived and designed the project. SY performed the experiments, analyzed the data, and wrote the manuscript. YW organized and saved the data. LK critically reviewed and curated the manuscript. All authors contributed to the article and approved the submitted version.

## Conflict of Interest

The authors declare that the research was conducted in the absence of any commercial or financial relationships that could be construed as a potential conflict of interest.

## Publisher’s Note

All claims expressed in this article are solely those of the authors and do not necessarily represent those of their affiliated organizations, or those of the publisher, the editors and the reviewers. Any product that may be evaluated in this article, or claim that may be made by its manufacturer, is not guaranteed or endorsed by the publisher.

## Footnotes

1
http://www.pacb.com/

2
http://circos.ca/

3
https://antismash.secondarymetabolites.org/

4
https://cge.cbs.dtu.dk/services/PathogenFinder/

5
https://ggdc.dsmz.de/victor.php#

6
https://cge.cbs.dtu.dk/services/ResFinder/

7
https://card.mcmaster.ca/analyze/rgi

## Supplementary Material

Supplementary Figure 1 Distribution of core, accessory, and unique genes on COG category.

Supplementary Figure 2 Phylogenetic tree of the intact phages identified in *W. confusa* strains.
