# Supplementary figures and images for: Complete Genome Sequence of Weissella confusa LM1 and Comparative Genomic Analysis
Source: Front Microbiol. 2021 Sep 28;12:749218. doi: 10.3389/fmicb.2021.749218 (PMC8506157; doi:10.3389/fmicb.2021.749218)

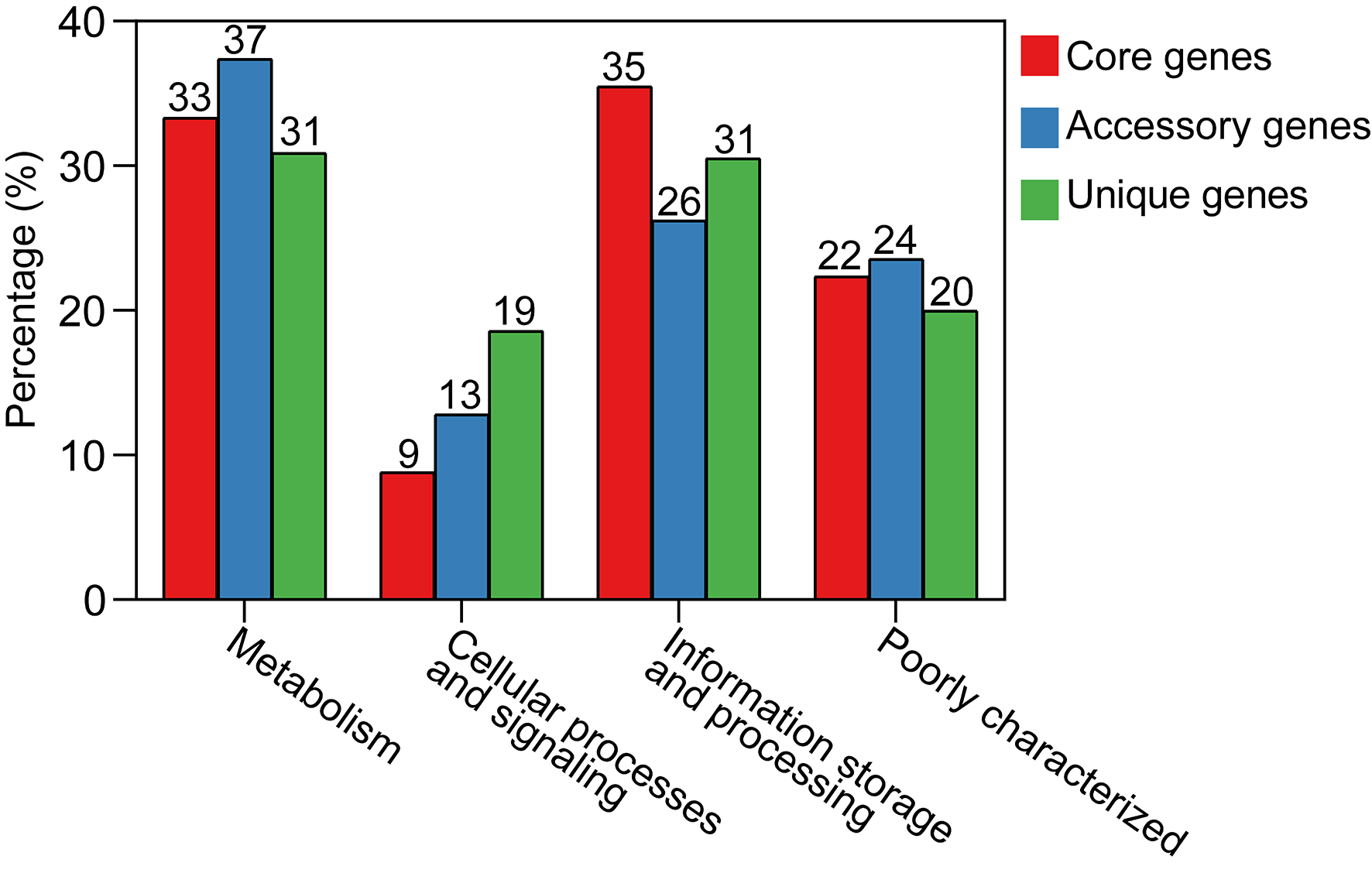

Supplement: Supplementary Figure 1 — Distribution of core, accessory, and unique genes on COG category. [file Image_1.TIF]

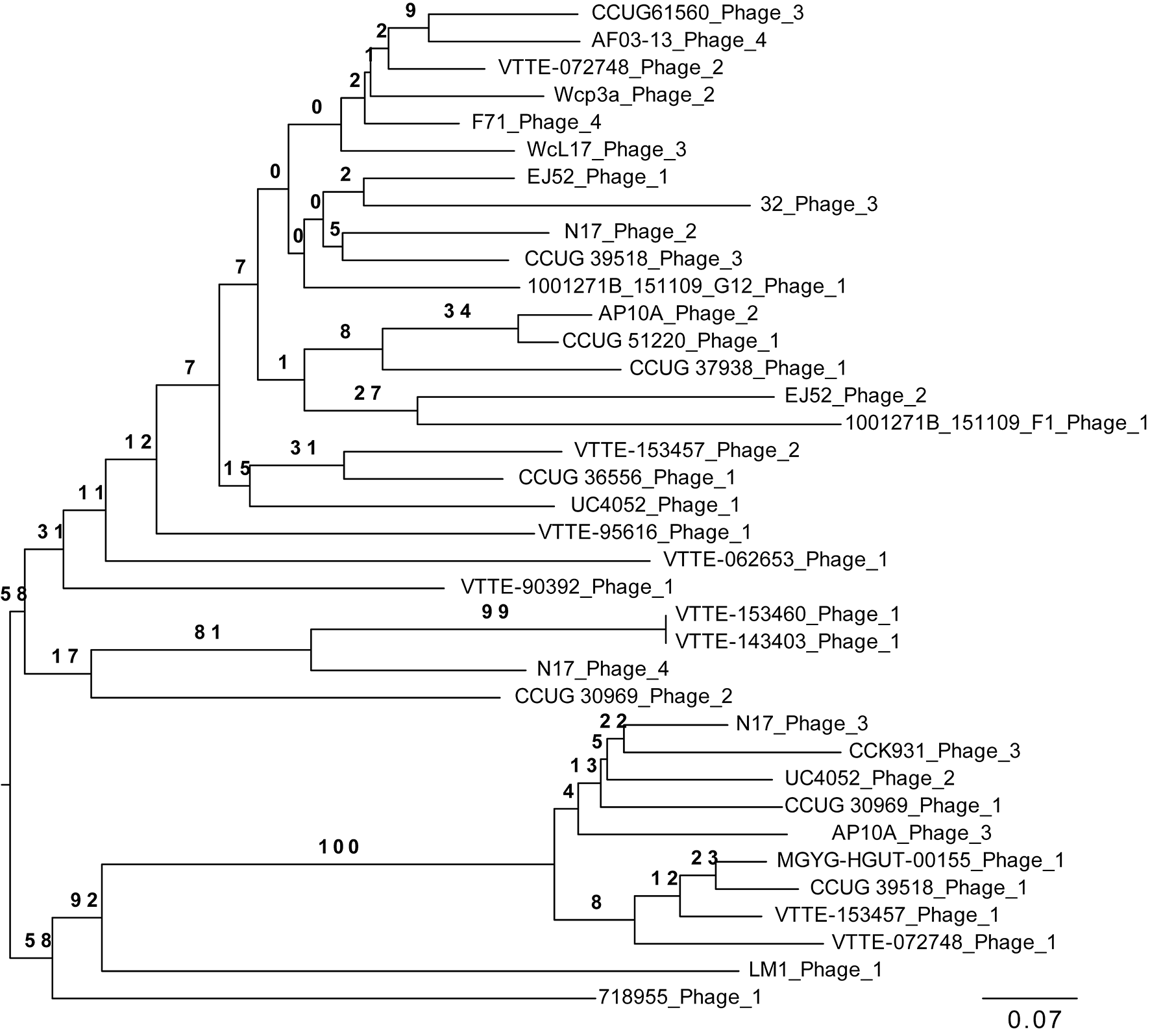

Supplement: Supplementary Figure 2 — Phylogenetic tree of the intact phages identified in W. confusa strains. [file Image_2.TIF]
